# Imaging of Skeletal Metastases in Myxoid Liposarcoma

**DOI:** 10.1155/2010/262361

**Published:** 2010-03-30

**Authors:** J. L. Noble, E. Moskovic, C. Fisher, I. Judson

**Affiliations:** ^1^Sarcoma Unit, The Royal Marsden NHS Foundation Trust, Fulham Road, London SW3 6JJ, UK; ^2^Department of Radiology, The Royal Marsden NHS Foundation Trust, Fulham Road, London SW3 6JJ, UK; ^3^Department of Histopathology, The Royal Marsden NHS Foundation Trust, Fulham Road, London SW3 6JJ, UK

## Abstract

Unlike other soft tissue sarcomas, myxoid/round cell liposarcoma (MRCL) has a tendency to spread to extrapulmonary sites but bone metastases are thought to be uncommon. In case reports, negative bone scintigraphy has been noted in patients with myxoid/round cell liposarcoma and bone metastases but the prevalence and optimal method of diagnosis of bone metastases in this common subtype of liposarcoma are unclear. In an attempt to answer these questions, data were obtained from a prospective database of patients with sarcoma, including MRCL, and the diagnostic imaging used was examined. A variety of imaging tools were used including plain X-rays, bone scintigraphy, computed tomography (CT), and magnetic resonance imaging (MRI). Eight patients (4.3%) developed skeletal metastases all of which were positive on MRI. Bone scintigraphy was negative in two out of four cases, CT was negative in six out of seven, and X-rays were negative in four. Radiography and CT measure mainly cortical bone involvement, whereas MRI examines bone marrow. When investigating patients with MRCL for bone pain, negative X-rays and bone scans do not rule out bone metastases. In our experience, MRI provides the most sensitive technique for the diagnosis of bone metastases in MRCL.

## 1. Introduction

Liposarcoma is one of the most common histologic types of soft tissue sarcoma. There are several subtypes of liposarcoma, of which myxoid liposarcoma represents about one third of cases [1]. Myxoid/round cell liposarcoma (MRCL) is associated with an unusual pattern of spread in that it has a predilection for extrapulmonary sites such as retroperitoneum, mediastinum, and other soft tissue sites [2–4]. The degree to which MRCL metastasizes to bone is not clear but the incidence in one series has been reported to be 17%, comprising 56% of all metastatic events [5]. Isolated case reports have also reported this phenomenon [6–8]. Screening for suspected bone metastases has classically been performed using bone scintigraphy but this may be inaccurate in tumours such as myeloma and renal cancer, in which conventional scintigraphy is inferior to plain X-ray [9, 10]. Following reports that bone scintigraphy also underdiagnoses metastatic bone disease in the case of MRCL [5, 6, 8], we performed this retrospective study to examine the prevalence of known bone metastases in our patient population and report our experience of diagnostic imaging with MRCL. 

## 2. Materials and Methods

Patients with a diagnosis of myxoid/round cell liposarcoma were identified from the prospective soft tissue sarcoma database. Between 1974 and 2006, 184 patients were diagnosed and treated. Well-differentiated, dedifferentiated, and pleomorphic liposarcomas were excluded. The diagnosis of MRCL was confirmed by a review of the histology in all cases at presentation. The diagnosis of MRCL was based on the presence of uniform round to oval shaped cells in a myxoid background, associated with a plexiform capillary network. Because of the lack of necrosis and minimal tumour cell pleomorphism, factors key to the grading of the majority of soft tissue sarcomas, grading in MRCL are instead based on the cellularity of the tumour and specifically the percentage of round cells noted on all individual sections. A two-tiered grading system has typically been employed where tumours with >5% round cells are considered high grade, and those with <5% round cells are considered low grade [3]. 

The medical records of all 184 patients with MRCL, including follow-up appointments, were reviewed. The reports of all imaging including plain X-rays, bone scintigraphy, computed tomography (CT), magnetic resonance imaging (MRI), and positron emission tomography (PET) were reviewed. All patients were followed up clinically with CT or chest X-ray surveillance for metastasis. The CT technique used was a conventional spiral scan for thorax abdomen and pelvis. If spinal disease was suspected or nerve root pain was being investigated, MRI was performed. Local recurrence was monitored by physical examination. Additional screening tests were requested if other sites of metastasis were suspected.

For the 8 patients identified with skeletal metastases, the actual imaging was reviewed where available. For the majority of spinal examinations, imaging was requested to investigate pain. All patients with suspected spinal metastases had a whole-spine MRI scan with T1- and T2- weighted sequences in the axial and sagittal planes. Where bone marrow abnormalities were observed on imaging but there was doubt concerning the existence of metastatic disease, a biopsy was performed and other diagnoses, such as multiple myeloma, were excluded by standard tests such as bone marrow biopsy and plasma immunoglobulin studies. Some, but not all, patients had bone scintigraphy and one patient had an FDG-PET scan.

## 3. Results

184 patients with a diagnosis of MRCL were identified from our prospective sarcoma database (Table 1). Eight (4.3%) of the 184 patients with MRCL were diagnosed with bone metastases, four of whom presented with back pain while the rest were discovered on routine imaging or investigation of tumour masses. In one patient, a spinal MRI was performed to investigate resectability of a paraspinal tumour, one patient had MRI of the trunk for ease of comparison with previous imaging and one patient with known abdominal disease had a deposit at L2 identified on an MRI. The spine was the first known site of metastasis in seven patients. Other sites of bone metastasis included sternum, femur, skull, and ribs. Four of the eight patients had bone scintigraphy which failed to demonstrate bone metastases in two cases and was equivocal in another. Seven of the eight patients had relevant CT scans of which six were negative for bone involvement and four patients had plain X-rays, all of which were negative for bone metastases. One patient underwent a PET scan which was negative for spinal metastases but only showed low-grade uptake in the primary tumour, hence the negative result is of uncertain significance.

These findings are illustrated by the following examples. 

**Figure 1:**
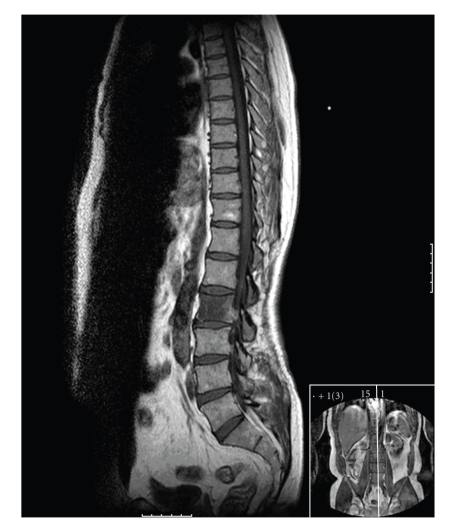
The MRI scan shows homogeneous isointense signal relative to skeletal muscle on T1-weighted images throughout L2 vertebra. The cortex is intact.

**Figure 2:**
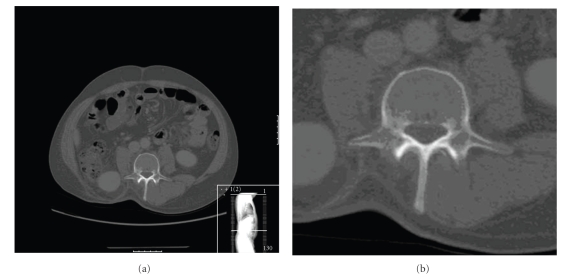
There is a right-sided paraspinal metastatic lesion. The bone windows on CT scan show very subtle lytic change in the vertebral body of L2 which was not considered significant at the time of reporting.

Patient 1 was diagnosed with MRCL in a paravertebral muscle which was excised with a marginal excision. He relapsed locally one year later and at this time an MRI scan showed tumour in the L2 vertebral body (Figure 1). Low signal was seen within the body of L2 on T1 corresponding to high signal on T2 in keeping with an intraosseous metastasis. A staging CT scan was performed showing no significant abnormality on bone windows at L2 or elsewhere (Figure 2). Plain X-rays and bone scan were not performed. The patient went on to receive chemotherapy with a good response but has since progressed. 

**Figure 3:**
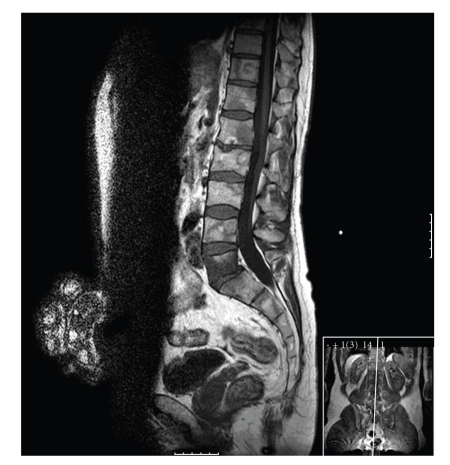
There is diffuse involvement of multiple vertebrae including L5 seen throughout the spine on T1-weighted imaging with both low and high signal.

**Figure 4:**
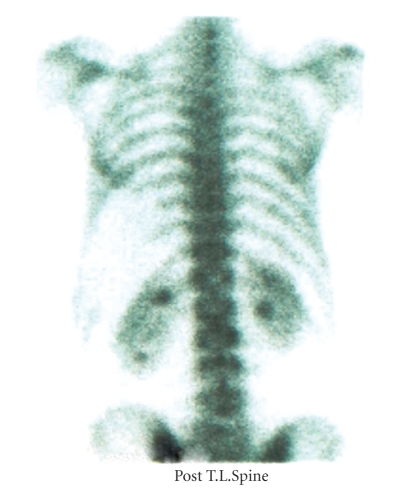
The synchronous bone scan shows no significant abnormality in the spine compared to the MRI scan.

**Figure 5:**
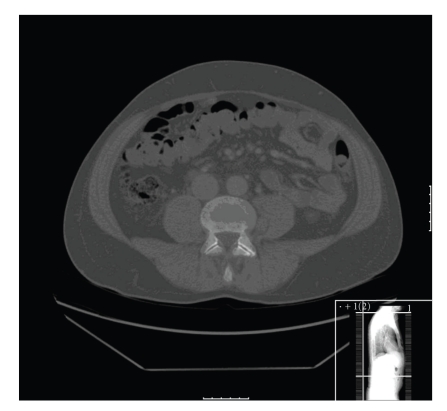
The CT image through L5 shows no significant abnormality on bone windows in contrast to the MRI scan at the equivalent level.

Patient 2 first presented in 1992 with MRCL in the left thigh which was excised. He relapsed locally on two occasions, treated by local excision. In 2005, during MRI staging for liver metastasectomy, multiple bone metastases were seen throughout the cervical, thoracic, lumbar spine, and sacrum (Figure 3). There was no evidence of cord compression. The patient was asymptomatic at the time. A bone scan, at this time, was negative for metastatic disease (Figure 4). The skeletal survey showed no lytic lesions and the CT also showed no evidence of tumour in the spine (Figure 5). The patient subsequently underwent chemotherapy and radiotherapy to sites of painful metastatic bone disease and died two years later.

## 4. Discussion

Liposarcoma is one of the commonest subtypes of soft tissue sarcoma constituting 9.8–18% of cases [1, 11]. The incidence peaks between 40 and 60 years and there is a slight male predominance [1]. The World Health Organization recognizes four subtypes: well-differentiated, de-differentiated, myxoid/round cell, and pleomorphic. The myxoid/round cell subtype is among the most prevalent and typically occurs in younger individuals with a peak in the 4th and 5th decades [12]. In contrast to other soft tissue sarcomas which tend to metastasize to the lung, liposarcoma has a propensity to spread to extrapulmonary sites [13, 14]. MRCL in particular tends to spread to other soft tissue sites including retroperitoneum, thorax, opposite extremity and other soft tissue sites before metastasizing to the lungs [2–4]. Skeletal metastases have also recently been reported in MRCL but the prevalence is unclear [12]. 

We identified 8 patients with skeletal metastases on radiographic criteria in a population of 184 patients with MRCL, that is, an incidence of 4.3%. Of these patients, metastatic bone disease was proven at spinal surgery in one case, thought likely on bone marrow aspiration cytology in one, and in one case was evident both clinically and on imaging in that the bone metastasis evolved into a large bone and soft tissue mass. The other patients all had other sites of metastatic disease at the time of diagnosis of metastatic bone disease. This is likely to be an underestimate of the true incidence of metastatic bone disease, since bone marrow involvement, in particular spinal metastases, can initially be asymptomatic, as we found. There have been several reports of the unreliability of various modes of imaging of bone metastases in MRCL. One of our cases has previously been reported emphasising the fact that the bone disease was only detectable by MRI [15]. Ishii et al. reported two cases of skeletal metastases from MRCL, both were undetectable using plain X-rays and bone scans but were detectable on MRI [6]. Similarly, Sheah et al., reported 12 patients with metastatic MRCL who were examined using CT, MRI, and PET [16]. CT showed mixed lytic and sclerotic foci, with bone destruction in advanced cases. MRI demonstrated fluid-like signal intensity with mild heterogeneous enhancement in cases of soft tissue metastases. In bone metastases, MRI showed avid heterogeneous enhancement. PET showed no significant FDG uptake for all metastases. They concluded that MRI was the most useful imaging modality for bone and soft tissue metastases. Similarly, there have been several other case reports of spinal metastases diagnosed using MRI which were undetectable on bone scintigraphy [7, 8, 17]. In our series, all plain X-rays were negative, two out of four bone scans were completely negative, and a PET scan was also negative. In the largest series reported to date, 33 of 230 patients with MRCL were found to have developed spinal metastases. Nine patients had bone scans, only three of which were positive in the spine and six patients had PET scans, only two of which were positive [5] which is consistent with our findings [5].

Bone scintigraphy is known to be more sensitive than plain X-rays in the detection of bone metastases in solid tumours. Scintigraphy may reveal bone metastases up to 18 months before they are apparent on radiography [18]. More than 50% of bone mineral content must be lost before metastasis is evident on X-ray [19], and cells growing in the marrow are less likely to be detected than those in the cortex [20]. But in certain conditions such as myeloma or lesions confined to the marrow, bone scans have low sensitivity [9]. It has been suggested that haematogenously seeded intramedullary metastases produce marrow replacement lesions detectable on MRI before any damage occurs in the adjacent bone [21]. The high contrast between fat and metastasis allows early demonstration of metastasis on MRI as soon as macroscopic lesions have developed in the marrow [22]. It is believed that isotope bone scans are negative in myeloma because myeloma cells not only promote osteoclast activity but also inhibit osteoblast differentiation [23]. In a series of 74 patients with metastatic bone disease from a number of different primary sites, it was found that bone scans were negative for all intramedullary lesions without cortical involvement shown on MR imaging. Bone scans were frequently positive for transcortical (71.3%) or subcortical involvement (33.8%). X-rays, CT, and bone scans mainly assess the cortex whilst MRI can detect disease in the bone marrow, where the early metastatic deposits occur. CT is sensitive in detecting subtle cortical invasion but less sensitive for medullary or bone marrow involvement [24]. In a retrospective comparison of bone scans and MR images in 35 patients, a higher sensitivity for MRI in detecting bone metastases was found compared to bone scans [25].

Techniques for whole body bone imaging using MRI have been investigated for the staging of patients with tumours, such as Ewing sarcoma, with a high incidence of metastatic bone disease [26]. MRI may produce fewer false positives than PET, while it may be somewhat less sensitive. In the context of MRCL, whole body MRI could be proposed as a staging investigation for the presence of metastatic bone disease. 

In conclusion, this is a retrospective review, with obvious limitations regarding the ability to identify asymptomatic patients with bone disease. In addition, we do not, as yet, have a policy to screen patients with metastatic MRCL using MRI. However, one may conclude that patients with this diagnosis who experience bone pain especially in the back, or other unexplained symptoms, should be suspected of having metastatic bone disease and for such patients MRI is the investigation of choice.

## Figures and Tables

**Table 1:** 

Age at diagnosis	Gender	Tumour grade*	Time to bone disease (yrs)	Sites of bone disease on MRI	CT#	X-ray#	Scintigraphy#	Other sites of disease
28	Male	low	5	Lumbar spine, sacrum	ND	ND	Neg	Soft tissue pelvis
44	Male	low	5.25	Spine, pelvis	Neg	Neg	Neg	Lungs
29	Male	High	0.6	Spine, skull, femur	Neg	Neg	Pos	Soft tissue abdomen
30	Female	High	6	Spine	Neg	ND	ND	Pleura
42	Male	High	11	Sternum, ilium	Pos sternum	ND	ND	Lung, soft tissue
34	Female	low	2.5	Dorsal spine	Neg	Neg	Equivoca	Abdo, multiple soft tissue
45	Male	High	0.75	Lumbar spine	Neg	ND	ND	Pleura, abdomen
72	Male	low	7.75	Dorsal spine	Neg	Neg	Neg	Paraspinal soft tissue

*<5% round cells = low grade, >5% = high grade.

# ND = not done.

Pos = confirmatory.

Neg = not confirmatory.
